# Efficient variant phasing utilizing a replication cycle reaction system

**DOI:** 10.1016/j.gimo.2025.103457

**Published:** 2025-09-19

**Authors:** Akihiko Mitsutake, Hiroyuki Ishiura, Takashi Matsukawa, Jun Mitsui, Shoji Tsuji, Tatsushi Toda

**Affiliations:** 1 Department of Neurology, Graduate School of Medicine, The University of Tokyo, Tokyo, Japan; 2 Department of Neurology, International University of Health and Welfare Mita Hospital, Tokyo, Japan; 3 Department of Neurology, Okayama University Graduate School of Medicine, Dentistry, and Pharmaceutical Sciences, Okayama, Japan; 4 Department of Precision Medicine Neurology, Graduate School of Medicine, The University of Tokyo, Tokyo, Japan; 5 Institute of Medical Genomics, International University of Health and Welfare, Chiba, Japan

**Keywords:** Autosomal recessive inheritance, Compound heterozygosity, Replication cycle reaction, Variant phasing

## Abstract

**Purpose:**

When 2 heterozygous variants are detected in autosomal recessive disease genes, determining whether they are in cis or in trans is essential. Subcloning polymerase chain reaction products or complementary DNA is limited by variant distance (up to 10 kb) and complementary DNA availability. Droplet digital polymerase chain reaction, effective up to 100 kb, faces probe design challenges. We used replication cycle reaction (RCR), which replicates large DNA fragments based on *E. coli* chromosome replication, to phase widely spaced heterozygous variants.

**Methods:**

Circular DNA molecules were formed by ligating CRISPR/Cas9-cleaved genomic fragments with an *oriC-AmpR* cassette, then amplified by RCR. Using a genomic DNA (gDNA) sample that is previously analyzed by long-read sequencing, we optimized reaction conditions (including gDNA to *oriC-AmpR* cassette ratios) and validated phasing accuracy via electrophoresis and Sanger sequencing. Finally, we applied this method to 7 patients harboring 2 heterozygous pathogenic variants (4.3-152 kb apart).

**Results:**

RCR amplified genomic regions up to 104 kb. Lower gDNA-to-cassette ratios favored monoallelic amplification, enabling straightforward phasing, whereas higher ratios yielded biallelic products requiring transformation-based allele separation. For variants 152 kb apart, an intervening single-nucleotide variant enabled phased reconstruction. Ultimately, RCR confirmed compound heterozygosity in all 7 patients.

**Conclusion:**

This method effectively phases multiple heterozygous variants across large genomic distances.

## Graphical abstract

**Figure:**
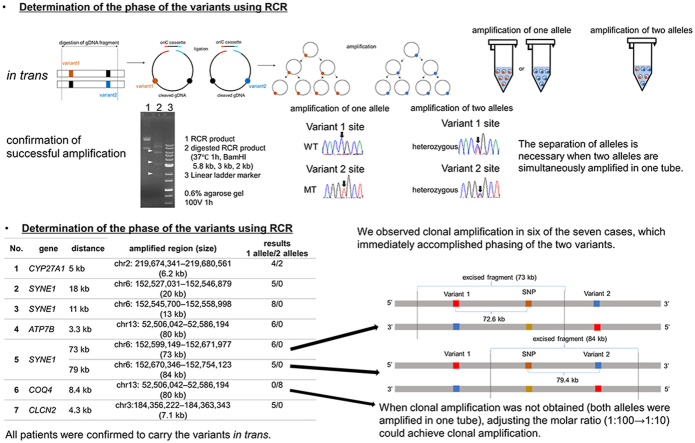

## Introduction

When 2 heterozygous variants are detected in genes for diseases with autosomal recessive inheritance, it is necessary to determine whether the 2 variants are located in cis or in trans—a process known as phasing. This determination is particularly important when 1 of the variants is a pathogenic variant and the other is a variant of uncertain significance. Accurate phasing can facilitate the reclassification of variant of uncertain significance and help resolve diagnostic ambiguities, as outlined in the ACMG/AMP variant classification guidelines.^1^

Although phasing is typically performed using the genomic DNAs (gDNAs) from the patient’s parents, we face difficulties when parental DNAs are unavailable. Subcloning of long-range polymerase chain reaction (PCR) products obtained from gDNAs is useful, but long-range PCR^2^ fails to amplify segments usually exceeding 10 kb. Subcloning of complementary DNA is another option, but its success largely depends on the availability of cells expressing the relevant messenger RNA. In addition, PCR amplification-based subcloning can occasionally yield inconsistent phase results, potentially attributed to incomplete elongation and mispriming on the heterologous allele producing PCR chimera.^3^ Droplet digital PCR is useful when these methods cannot be applied.^4^ This method isolates gDNA into 30,000 to 50,000 droplets in such a way that a single DNA molecule in each droplet is amplified with allele-specific fluorescence-labeled probes. In principle, an appropriate combination of allele-specific fluorescent probes will produce double-positive droplets when DNA fragments containing physically linked variants are present in the same droplets. This method can be applied when the distance between variants is up to 100 kb,^4^ although designing and optimizing allele-specific probes sometimes remain challenging. Although long-read sequencing provides an alternative approach for phasing, its implementation remains constrained by high costs and the need for specialized infrastructure. These resource demands make it challenging for many laboratories to adopt long-read sequencing as a routine method.

**Figure 1:**
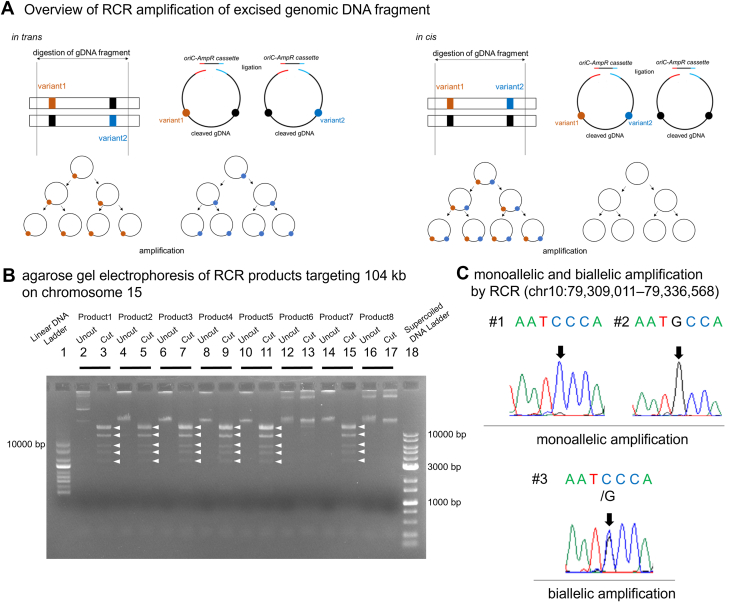
**Overview of RCR amplification and gel electrophoresis of RCR products.** A. Overview of RCR amplification of excised genomic DNA fragment. in trans: gDNAs were cleaved using CRISPR/Cas9, generating cleaved segments with 2 heterozygous variants. The cleaved gDNA fragments were ligated to the *AmpR*-*oriC* cassette, forming circular DNA molecules. Two distinct types of circular DNA molecule, each containing 1 of the variants, were produced. Subsequently, the circular DNA molecules were amplified by RCR. in cis: gDNAs were cleaved using CRISPR/Cas9, generating cleaved segments with 2 heterozygous variants. The cleaved gDNA fragments were ligated to the *AmpR-oriC* cassette, forming circular DNA molecules. Two types of circular DNA molecule, 1 containing both variants and the other without either variant, were produced. Subsequently, the circular DNA molecules were amplified by RCR. B. Agarose gel electrophoresis of RCR products targeting 104 kb on chromosome 15. Eight independent RCR products of 104 kb were loaded onto a 1% agarose gel. Undigested RCR products and those digested with SacI were assessed. Successful RCR amplification was observed in products 1, 2, 3, 4, 5, and 7 through the examination of digested RCR products sizes (16 kb, 14 kb ×2, 9.2 kb ×2, 6.1 kb, 4.8 kb, and 3.5 kb, respectively). Lanes 1, size standard marker (1 kb DNA ladder); 2, undigested RCR product 1; 3, digested RCR product 1; 4, undigested RCR product 2; 5, digested RCR product 2; 6, undigested RCR product 3; 7, digested RCR product 3; 8, undigested RCR product 4; 9, digested RCR product 4; 10, undigested RCR product 5; 11, digested RCR product 5; 12, undigested RCR product 6; 13, digested RCR product 6; 14, undigested RCR product 7; 15, digested RCR product 7; 16, undigested RCR product 8; 17, digested RCR product 8; and 18, supercoiled DNA ladder. C. Monoallelic and biallelic amplifications by RCR (chr10:79,309,011-79,336,568). The region chr10:79,309,011-79,336,568 is amplified by RCR and subsequently sequenced. One allele is amplified in products 1 and 2, whereas 2 alleles are amplified in product 3. RCR, replication cycle reaction.

In this study, we present the replication cycle reaction (RCR) as a method to bridge the gap between PCR-based subcloning and long-read sequencing. Although PCR-based subcloning can be hampered by large genomic distances, and long-read sequencing remains both costly and infrastructure-intensive, RCR provides a cost-effective yet powerful strategy for phasing variants spanning genomic regions of up to approximately 150 kb. RCR is the reconstitution of the entire replication cycle of *E. coli in vitro*.^5^ RCR propagates amplified circular DNA in an isothermal reaction.^5^ This method can amplify a very large circular DNA carrying the *oriC* sequence up to 0.2 Mb. Furthermore, it is characterized by a high accuracy of replication, approximately ∼1.2 × 10^−8^ per base per replication cycle. By combining the *oriC* sequence and the excised gDNA fragment, we hypothesized that it would be possible to amplify genomic regions too large to be amplified by conventional PCR (Figure 1A). To accomplish the amplification of a large genomic DNA fragment carrying 2 heterozygous variants, we used RCR and tried to determine whether the heterozygous variants are located in cis or in trans. First, we optimized the conditions for RCR amplification of the excised gDNA fragment. We then evaluated the utility of RCR for variant phasing using DNA samples previously analyzed by long-read sequencing and successfully confirmed accurate phasing. Finally, we applied this approach to samples in actual clinical practice.

## Materials and Methods

### Samples

We first used a gDNA sample (#9565) previously analyzed by long-read sequencing to optimize RCR conditions and evaluate phasing accuracy. After confirming the feasibility and reliability of RCR with this sample, we then applied the method to 7 additional clinical samples carrying 2 heterozygous variants each. This study was approved by the Institutional Review Board of the Graduate School of Medicine, The University of Tokyo. Written informed consent was obtained from all the patients.

### Digestion of gDNA

gDNAs were extracted from peripheral blood leukocytes following a standard procedure. Extracted gDNAs were digested using a CRISPR/Cas9 system. CHOPCHOP (https://chopchop.cbu.uib.no/) was used to design the CRISPR RNA sequence, a 20-nucleotide sequence upstream of the PAM sequence (5′-NGG-3′). Then, we synthesized a single guide RNA (sgRNA) from target-specific oligonucleotides with an EnGen sgRNA Synthesis Kit (New England Biolabs, #E2233S) following the manufacturer’s instructions. After the purification of sgRNA using an RNeasy Plus Mini Kit (QIAGEN Venlo), 10 μg of gDNA was cleaved with Cas9 Nuclease, *S. Pyogenes* (New England Biolabs. #M0386S). A total of 5 μL of NEBuffer r3.1, 5 μL of sgRNA (5 μM), 1 μL of Cas9 nuclease (20 μM), and nuclease-free water were mixed in a total volume of 50 μL. The mixture was incubated for 1 hour at 37 °C and then for 20 min at 65 °C. The cleaved gDNA was purified with AmPure XP (Agencourt Bioscience Corp). The quality of gDNA was evaluated using a 4150 TapeStation (Agilent Technologies).

### RCR amplification of gDNA fragment

An *oriC* cassette contained in the OriCiro Cell-Free Cloning System (OriCiro Genomics) was subcloned into pBR322 downstream of the ampicillin resistance gene using an In-Fusion HD Cloning Kit (Takara Bio). The nucleotide sequence of the plasmid is shown in Supplemental Methods 1.

With the vector as a template, the region containing the cassette with *rop*, *ori*, *AmpR*, and *oriC* cassettes (*oriC*-*AmpR* cassette) was amplified by PCR using primer pairs containing 60 nt overlapping sequences with the target sequence located on the 5′-side, followed by PCR product purification. This procedure was conducted for the subsequent transformation of RCR products. The detailed PCR procedure and the primer sequences are described in Supplemental Methods 2. Then, we ligated the cleaved gDNA fragment and PCR-amplified *oriC*-*AmpR* cassette using the 2× RA mix contained in the OriCiro Cell-Free Cloning System to form a circular DNA molecule. The reaction conditions are as follows: the cleaved gDNA fragment and *oriC-AmpR* cassette were mixed in a total of 2.4 μL at various molar ratios of gDNA to the *oriC-AmpR* cassette (1:10, 1:100, 1:1000, and 1:10,000). The amount of gDNA input was fixed at 100 ng (1.4 μL), and various amounts of *oriC-AmpR* cassette (1.6 pg, 16 pg, 160 pg, and 1.6 ng) were added to a total volume of 1.1 μL. After adding 2.5 μL of 2× RA Mix, the mixture was incubated for 1 hour at 42 °C to form circular DNA molecules by an enzyme-based annealing mechanism using 2× RA Mix.^4^ Afterward, we mixed 0.5 μL of the product with 1 μL of 5× Buffer I, 1 μL of 5× Buffer II, 0.5 μL of 10× RE mix, and 2 μL of nuclease-free water. The resulting mixture was then incubated for 16 hours at 33 °C for the amplification of circular DNA molecules by RCR. The successful amplification by RCR was confirmed by agarose gel electrophoresis (0.6%) of RCR products (100 V, 1 h) after digestion with specific restriction enzymes.

### The number of amplified alleles in RCR and the accuracy of phasing

In this experiment, we used a gDNA sample (#9565) already analyzed by long-read sequencing. Genome sequencing was performed using the PacBio RSII long-read platform (Pacific Biosciences) with gDNA extracted from lymphoblastoid cells.^6^ Circular consensus sequence reads were generated from the subreads using the ccs application SMRT Link version 6.0.0.47841 provided by PacBio (https://ccs.how/). The reads were aligned to GRCh38/hg38 using Minimap2.^7^ The result was visualized using Integrated Genome Viewer (version 2.8.6).^8^

First, we amplified a genomic region of approximately 100 kb (chr15:44,852,987-44,956,565; 104 kb, NC_000015.9, GRCh37) to assess whether amplification by RCR is feasible. This region encompasses the entire *SPG11* gene (HGNC:11226). The molar ratio of gDNA to the *oriC-AmpR* cassette was fixed at 1:100. Next, we amplified 8 regions containing heterozygous single-nucleotide variants (SNVs) that had been phased by long-read sequencing. The regions and corresponding SNVs are shown in Table 1. These genomic regions were digested using sgRNA and subsequently amplified by RCR. Amplification was performed in 5 independent tubes each for regions 1-5 and in 8 independent tubes for region 6. We then analyzed the sequences at these SNV sites in the amplified products to determine how many alleles had been amplified. Regions 1, 2, 5, and 6 contained multiple heterozygous SNVs, enabling us to assess the consistency of variant phasing between RCR-based method and long-read sequencing. Specifically, we compared the phases derived from single-allele RCR amplification products with the established phases obtained through long-read sequencing data.

**Table 1:** Amplified genomic regions and heterozygous SNVs

No.	Region	Size (kb)	SNV1	SNV2	SNV3
1	chr14:88,450,095-88,455,957	5.9	NC_000014.8:g.88453186A>G	NC_000014.8:g.88453278T>G	-
2	chr5:149,503,216-149,513,393	10.2	NC_000005.9:g.149511792A>G	NC_000005.9:g.149512042G>A	-
3	chr10:79,309,011-79,336,568	27.6	NC_000010.10:g.79316010C>G	-	-
4	chr8:655,279-657,586	2.3	NC_000008.10:g.656511C>T	-	-
5	chr13:52,524,496-52,538,815	9.2	NC_000013.9:g.52515354A>G	NC_000013.9:g.52520704C>A	NC_000013.9:g.52523808C>T
6	chr13:52,580,371-52,594,755	14.3	NC_000013.9:g.52524560T>C	NC_000013.9:g.52531960T>C	-

*SNV*, single-nucleotide variant.

### Determination of the optimal molar ratio of gDNA to *oriC* cassette

Various molar ratios of gDNA to the *oriC-AmpR* cassette were evaluated to determine the optimal conditions for the ligation step. Additionally, the accuracy of variant phasing using the RCR method was assessed through comparative analyses with long-read sequencing data.

We used a gDNA sample (#9565) that had previously been analyzed by long-read sequencing as described above. Regions 1-3 listed in Table 1 were selected, digested with sgRNA, and then amplified by RCR. Heterozygous SNVs located in these regions are listed in Table 1. Each sample was amplified by RCR in 8 independent tubes for each molar ratio. Successful RCR amplification was confirmed by electrophoresis after digestion with specific restriction enzymes. After confirming successful amplification, the RCR products were then subjected to direct nucleotide sequence analysis to determine whether 1 or 2 alleles were amplified in each tube. Regions 1 and 2 each harbored 2 SNVs, and we determined the phase of these SNVs. To assess the accuracy of variant phasing using the RCR method, we used RCR products in which only 1 allele was amplified and compared them with long-read sequencing data.

### Direct nucleotide sequence analysis of RCR products

The obtained RCR products were purified using ExoSAP-IT for PCR Product Clean-Up (Affymetrix), with 1 cycle of 30 min at 37 °C followed by another cycle of 15 minutes at 80 °C. The purified RCR products were subjected to nucleotide sequence analysis using the ABI PRISM BigDye 3.1 terminator method (Applied Biosystems) and the ABI PRISM 3100 Genetic Analyzer (Applied Biosystems). The sequences of the primers are shown in Supplemental Methods 3.

### Analysis of gDNAs from patients carrying 2 heterozygous variants in a clinical setting

In this experiment, we analyzed 7 gDNA samples already analyzed by exome sequencing. gDNA samples were from 7 patients carrying 2 heterozygous variants in *SYNE1* (HGNC:17089), *CYP27A1* (HGNC:2605), *ATP7B* (HGNC:870), *COQ4* (HGNC:19693), or *CLCN2* (HGNC:2020). The variants are shown in Table 2. All of the variants were classified as pathogenic or likely pathogenic according to the ACMG/AMP variant classification guidelines.^1^ The distances between the 2 variants ranged from 4.3 to 152 kb (Table 2).

**Table 2:** Summary of heterozygous variants, distances between variants in each patient, and RCR amplification results

No	Gene (HGNC) Transcript	Variant 1 ACMG Classification^1^	Variant 2 ACMG Classification^1^	Distance	Amplified Region (GRCh37)	Amplified Alleles 1 Allele/2 Alleles
1 #10614	*CYP27A1* (2605) NM_000784.4 (NP_000775.1)	c.410G>A, p.(Arg137Gln) NC_000002.11:g.219674454G>A Pathogenic (PS4, PM2, PM3, PM5)	c.1421G>A, p.(Arg474Gln) NC_000002.11:g.219679425G>A Pathogenic (PS3, PS4, PM2, PM5)	5 kb	chr2:219,674,341-219,680,561 (6.2 kb)	4/2
2 #3946	*SYNE1* (17089) NM_033071.5 (NP_149062.2)	c.21250C>T, p.(Arg7084Ter) NC_000006.11:g.152545688G>A Pathogenic (PVS1, PS4, PM2)	c.22622_22623del, p.(Lys7541SerfsTer28) NC_000006.11:g.152527489_152527490del Likely pathogenic (PVS1, PM2)	18 kb	chr6:152,527,031-152,546,879 (20 kb)	5/0
3 JCAT0069	*SYNE1* (17089) NM_033071.5 (NP_149062.2)	c.19943_19944del, p.(Glu6648GlyfsTer8) NC_000006.11:g.152557995_152557996del Likely pathogenic (PVS1, PM2)	c.21100C>T, p.(Arg7034Ter) NC_000006.11:g.152546894G>A Likely pathogenic (PVS1, PM2)	11 kb	chr6:152,545,700-152,558,998 (13 kb)	8/0
4 #11528	*ATP7B* (870) NM_000053.4 (NP_000044.2)	c.2810del, p.(Val937GlyfsTer5) NC_000013.10:g.52523853del Pathogenic (PVS1, PS4, PM2)	c.2975C>T, p.(Pro992Leu) NC_000013.10:g.52520505G>A Pathogenic (PS4, PS3, PM2, PP3, PP5)	3.3 kb	chr13:52,506,042-52,586,194 (80 kb)	6/0
5 C0438	*SYNE1* (17089) NM_033071.5 (NP_149062.2)	c.4640_4643dup, p.(Gln1548HisfsTer19) NC_000006.11:g.152751684_152751687dup Likely pathogenic (PVS1, PM2)	c.11576A>C, p.(Lys3859Thr) NC_000006.11:g.152671865T>G Benign (BA1)	79 kb	chr6:152,670,346-152,754,123 (84 kb)	6/0
		c.11576A>C, p.(Lys3859Thr) NC_000006.11:g.152671865T>G Benign (BA1)	c.18325C>T, p.(Gln6109Ter) NC_000006.11:g.152599259G>A Likely pathogenic (PVS1, PM2)	73 kb	chr6:152,599,149-152,671,977 (73 kb)	5/0
6 #12162	*COQ4* (19693) NM_016035.5 (NP_057119.3)	c.238C>T, p.(Arg80Cys) NC_000009.11:g.131087457C>T VUS (PM2, PP3)	c.718C>T, p.(Arg240Cys) NC_000009.11:g.131095844C>T Likely pathogenic (PS4, PM2, PM5)	8.4 kb	chr9:131,084,924-131,096,154 (11 kb)	0/8
7 #12302	*CLCN2* (2020) NM_004366.6 (NP_004357.3)	c.61dup, p.(Leu21ProfsTer27) NC_000003.11:g.184079209dup Pathogenic (PVS1, PM2, PM3)	c.983+2T>A NC_000003.11:g.184074968A>T Pathogenic (PVS1, PS3, PM2)	4.3 kb	chr3:184,074,010-184,081,131 (7.1 kb)	5/0

For patient 1, RCR products in which 2 alleles were amplified in a single tube were transformed in HST08 Premium Competent Cells (Takara Bio). The resultant colonies were picked, and plasmid DNA was extracted using a QIAprep Spin Miniprep Kit (QIAGEN). Direct nucleotide sequence analysis of plasmid DNA was performed.

## Results

### The number of amplified alleles in RCR

We used a gDNA sample (#9565) already analyzed by long-read sequencing. First, we attempted to amplify a genomic region of approximately 100 kb, fixing the molar ratio of gDNA to the *oriC-AmpR* cassette at 1:100. We successfully amplified the chr15:44,852,987-44,956,565 (104 kb, NC_000015.9, GRCh37) (Figure 1B). Although we initially expected amplification of both alleles (biallelic amplification) in each reaction, we occasionally observed amplification of only 1 allele (monoallelic amplification) (Figure 1C).

We then evaluated whether 1 or both alleles were amplified using the RCR method for regions 1-6. Amplification was performed 5 times for regions 1-5 and 8 times for region 6. As shown in Table 3, monoallelic amplification was achieved in 8 amplicons, whereas biallelic amplification was observed in 18 amplicons. We were able to determine the phase from amplicons exhibiting monoallelic amplification in regions 1, 5, and 6. None of these amplicons showed inconsistent results. These results were consistent with those obtained from long-read sequencing (Table 3). Moreover, whether amplification was monoallelic or biallelic did not appear to depend on amplicon size.

**Table 3:** RCR amplification results for the genomic regions listed in Table 1

No.	Amplified Alleles 1 Allele/2 Alleles	Phase Determined by RCR	Phase Determined by Long-read Sequencing
1	2/1	AT/GG	AT/GG
2	0/5	Not determined	AG/GA
3	0/3	NA	NA
4	3/0	NA	NA
5	1/4	ACC/GAT	ACC/GAT
6	2/5	TT/CC	TT/CC

*RCR*, replication cycle reaction.

### Optimization of molar ratio of gDNA to *oriC-AmpR* cassette and RCR success rate

We used a gDNA sample (#9565) already analyzed by a long-read sequencing.

We examined whether the molar ratios of gDNA to the *oriC-AmpR* cassette at the ligation step affected the rate of successful RCR amplification and biallelic/monoallelic amplification. Thus, regions 1-3 shown in Table 1 were amplified at various molar ratios from 1:10 to 1:10,000. As a result, we found that the molar ratio of gDNA to the *oriC-AmpR* cassette considerably affected the rate of successful RCR amplification (Table 4). When the input *oriC-AmpR* cassette amount was low (molar ratio of 1:10, 1.6 pg), the rate of successful RCR amplification was low (4/24), and only 1 allele was amplified (4/4). In contrast, when the input *oriC-AmpR* cassette amount was high (molar ratio of 1:1000, 1.6 ng), the rate of successful RCR amplification was high (20/24), and 2 alleles were amplified (18/20).

**Table 4:** Effect of the gDNA to *oriC-AmpR* cassette molar ratio on RCR amplification success rate and amplified alleles

gDNA:oriC	Region 1		Region 2		Region 3	
	Success rate of RCR Amplification	Amplified Alleles 1 Allele/2 Alleles	Success rate of RCR Amplification	Amplified Alleles 1 Allele/2 Alleles	Success rate of RCR Amplification	Amplified Alleles 1 Allele/2 Alleles
1:10	0/8	0/0	1/8	1/0	3/8	3/0
1:100	1/8	1/0	2/8	2/0	7/8	3/4
1:1000	3/8	2/1	8/8	0/8	7/8	0/7
1:10,000	4/8	2/2	8/8	0/8	8/8	0/8

*RCR*, replication cycle reaction.

To assess the accuracy of variant phasing, we compared the phasing results obtained from RCR products generated by monoallelic amplification with those from long-read sequencing data in regions 1 and 2. The results are shown in Table 5. We were able to determine the phases of the variants in each region, which were consistent with those obtained by long-read sequencing. None of these amplicons showed inconsistent results.

**Table 5:** Comparison of phase determination by RCR and long-read sequencing in the genomic regions listed in Table 4

No	Phase Determined by RCR	Phase Determined by Long-read Sequencing
1	AT/GG	AT/GG
2	AG/GA	AG/GA

*RCR*, replication cycle reaction.

### Determination of the phase of pathogenic variants by RCR amplification in a clinical setting

**Figure 2:**
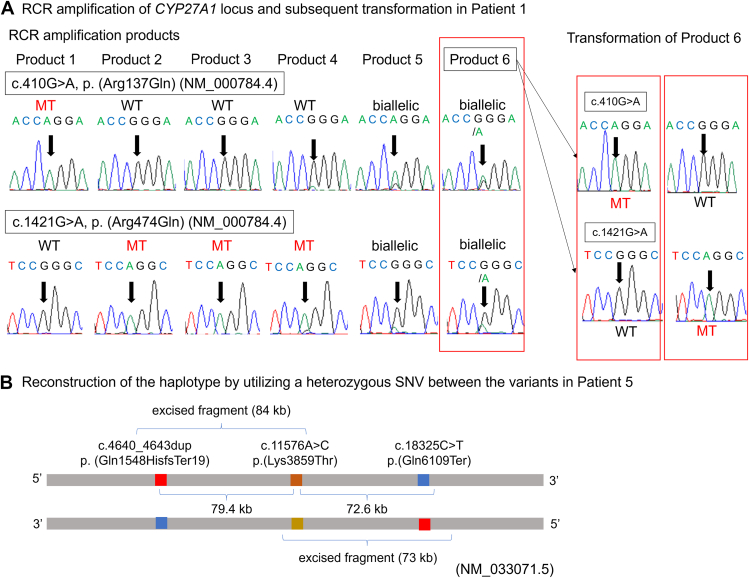
**RCR amplification and determination of the phase.** A. RCR amplification of *CYP27A1* locus and subsequent transformation in patient 1. Exome sequencing identified 2 heterozygous missense variants in *CYP27A1* (NM_000784.4): c.410G>A [p.(Arg137Gln)] and c.1421G>A [p.(Arg474Gln)]. A previous analysis by PCR amplification-based subcloning yielded inconsistent phase results. RCR was utilized to determine the phase of this patient. Six RCR products were prepared, and direct nucleotide sequence analysis was performed. Only 1 allele was amplified in products 1 to 4. Two alleles were amplified in products 5 and 6, for which Sanger sequencing suggested some skewed amplification. Product 6 was transformed in *E. coli* and resultant colonies were picked. The successful transformation was confirmed by the electrophoresis of plasmid DNA, and direct nucleotide sequence analysis showed consistent phasing results. B. Reconstruction of the haplotype by utilizing a heterozygous SNV between the variants in patient 5. We were unable to amplify the entire region containing 2 heterozygous variants (152 kb) probably because of its large size. Exome sequencing showed that there was a heterozygous SNV located 78 kb away from the variant [c.11576A>C, p.(Lys3859Thr) (NM_033071.5)], and we utilized this SNV to reconstruct the haplotype. RCR, replication cycle reaction; SNV, single-nucleotide variant.

We used gDNA samples from patients already analyzed by exome sequencing. In these patients, multiple heterozygous variants were detected in genes for diseases with autosomal recessive inheritance. The variants are listed in Table 2. All the variants were classified as pathogenic or likely pathogenic. Nucleotide sequence analysis of the RCR products was conducted to determine the phase in 7 cases that require the confirmation of compound heterozygosity. We observed the monoallelic amplification of 1 of 2 variants in 6 of the 7 cases (Table 2), for which the phasing of the 2 variants was immediately accomplished except for patient 6 (Figure 2A, Supplemental Figure 1A-F).

For patient 1, 4 RCR products showed monoallelic amplification (only 1 allele was amplified in a tube), and in the other 2 PCR products (products 5 and 6), DNA molecules originating from both alleles were amplified (Figure 2A). The phase was determined using 4 RCR products with monoallelic amplification, but we further examined whether alleles could be separated by transforming the RCR products with biallelic amplification. Both alleles were almost equally amplified in product 6, and this product was transformed. Direct nucleotide sequence analysis of the plasmid DNA confirmed the phasing (Figure 2A). Thus, the transformation of the RCR products enabled us to separate the alleles and determine the phase.

For patient 5, the distance between 2 variants was 152 kb (Figure 2B). Although we first tried to amplify the region containing both variants (152 kb), it was impossible to amplify the entire region. Exome sequencing showed that there was a heterozygous SNV located 78 kb away from the variant [c.11576A>C, p.(Lys3859Thr) (NM_033071.5)]. We were also able to reconstruct the haplotype by utilizing this SNV (Figure 2B). We were able to amplify the region spanning variant 1 and the SNV and the region spanning the SNV and variant 2 separately, which eventually enabled us to determine the phase.

For patient 6, 2 alleles were simultaneously amplified in each tube by RCR. The molar ratio of the gDNA:*oriC-AmpR* cassette was changed to 1:10 to facilitate monoallelic amplification. We achieved monoallelic amplification at this molar ratio, resulting in the confirmation of the compound heterozygosity (Supplemental Figure 1E). The reason why all the amplicons showed biallelic amplification remains unclear. This phenomenon underscores the need for empirical titration in each genomic region.

## Discussion

In this study, we successfully amplified excised gDNA fragments up to 104 kb using RCR. The analysis of RCR products proved to be valuable for phasing multiple heterozygous variants. In fact, we successfully determined the phase of the variants in 7 patients with various diseases with autosomal recessive inheritance.

Increasing the amount of the *oriC*-*AmpR* cassette increases the rate of successful RCR amplification; however, this also increases the possibility of 2 alleles being amplified in a single tube. Conversely, reducing the amount of the *oriC-AmpR* cassette decreases the rate of successful RCR amplification but lowers the possibility of both alleles being amplified in a single tube, which means that there is an increased possibility of monoallelic amplification. Thus, even when amplifying the same region, the number of amplified alleles varied depending on the conditions of the ligation step. Of note, achieving a monoallelic or skewed amplification was useful for the prompt phasing of the 2 variants. Maintaining a low concentration of the *oriC-AmpR* cassette is preferable for monoallelic amplification. However, if this proves challenging, increasing its concentration may improve the rate of successful RCR amplification. Furthermore, our method enables the transformation of RCR products in *E. coli*, which is also useful for allele separation (Figure 2A).

In patient 1, a previous study of PCR amplification-based subcloning yielded inconsistent phasing results, presumably because of incomplete elongation and mispriming on the heterologous allele. On the other hand, RCR products with monoallelic amplification showed consistent phase results in 16 individual RCR amplification processes.

The design of sgRNA necessary for cleaving gDNA varies depending on the region of interest. To mitigate variability in cutting efficiency and minimize off-target effects, we use bioinformatics tools such as CHOPCHOP for sgRNA design, selecting sgRNAs that balance optimal on-target activity with minimal predicted off-target interactions. However, when an entire gene can be amplified by RCR, the same sgRNA can be used for that gene, regardless of the variants of interest within the gene. In this study, we were able to design the sgRNA for the RCR amplification of the entire *ATP7B* as demonstrated for patient 4. Thus, the phasing of any variants in this gene can be performed using the same sgRNA and *oriC-AmpR* cassette for patients harboring multiple heterozygous variants in *ATP7B*.

Compared with other methods for phasing, the RCR method is simple and effective. It presents several key advantages: First, no chimeric sequences were observed as in PCR-based methods. All amplicons showed consistent results. Second, unlike ddPCR, it does not require specialized devices. Third, sgRNA design for RCR is notably more flexible than the design and optimization required for allele-specific probes in ddPCR. Fourth, although long-read sequencing is an alternative for phasing, it remains costly. Finally, RCR provides actual DNA fragments that are suitable for downstream functional analyses, such as splicing assays.

Nevertheless, the RCR method has some limitations. In contrast to long-read sequencing, which supports broader genomic distances, RCR struggles to amplify regions larger than approximately 150 kb. Furthermore, successful monoallelic amplification in RCR requires careful optimization of the gDNA to *oriC-AmpR* cassette molar ratio, reducing the uniformity and automation possible with long-read sequencing. In particular, inconsistent optimized gDNA to *oriC-AmpR* cassette molar ratios across different genomic regions make this method more complicated. Currently, we recommend starting with a 1:100 ratio and then adjusting based on the outcomes because this approach provides an effective balance between efficiency and practicality.

Despite these constraints, RCR method proves invaluable when precise phasing of targeted regions is required without access to parental samples or long-read sequencing resources, offering a cost-effective and efficient alternative for reliable phase determination and functional assays. Moreover, RCR uniquely provides actual DNA fragments, making it an excellent choice for downstream functional analyses, such as splicing assays, in which having intact DNA is crucial for accurate results. One potential clinical application of RCR-based phasing is in preimplantation genetic testing, particularly when only the proband’s DNA is available. Conventional phasing methods typically require parental samples, but RCR enables accurate phasing of large genomic regions using DNA from a single individual. This approach is particularly promising for identifying disease-associated variant combinations in autosomal recessive disorders during preimplantation genetic testing. Moreover, the efficiency and scalability of the method may support its integration into clinical workflows, improving precision in complex diagnostic settings.

In summary, we established an RCR method using OriCiro technology, enabling long-range amplification. Depending on the molar ratio of the *oriC-AmpR* cassette to gDNA, we found that the success rate varied, and monoallelic or biallelic amplification occurred. Determining the phase is one of the burdens in the diagnosis of autosomal recessively inherited diseases, particularly when DNA samples of parents are unavailable. Thus, we propose a new method for phase determination in this study. In the future, further optimization of the ligation conditions, including advanced enzyme cocktails or refined sgRNA designs, may extend the maximum amplification range beyond 150 kb and pave the way for simplified workflow. These improvements could solidify RCR’s role as a versatile method for variant phasing in both research and clinical settings.

## Data Availability

Data are available on request from the authors.

## Conflict of Interest

The authors declare no conflicts of interest.
