# Supplementary material for: Efficient variant phasing utilizing a replication cycle reaction system
Source: Genet Med Open. 2025 Sep 19;3:103457. doi: 10.1016/j.gimo.2025.103457 (PMC12590433; doi:10.1016/j.gimo.2025.103457)
Supplement: Supplemental Methods [file mmc1.pdf]

Supplementary Methods 1. pBR322 plasmid with oriC cassette sequence.

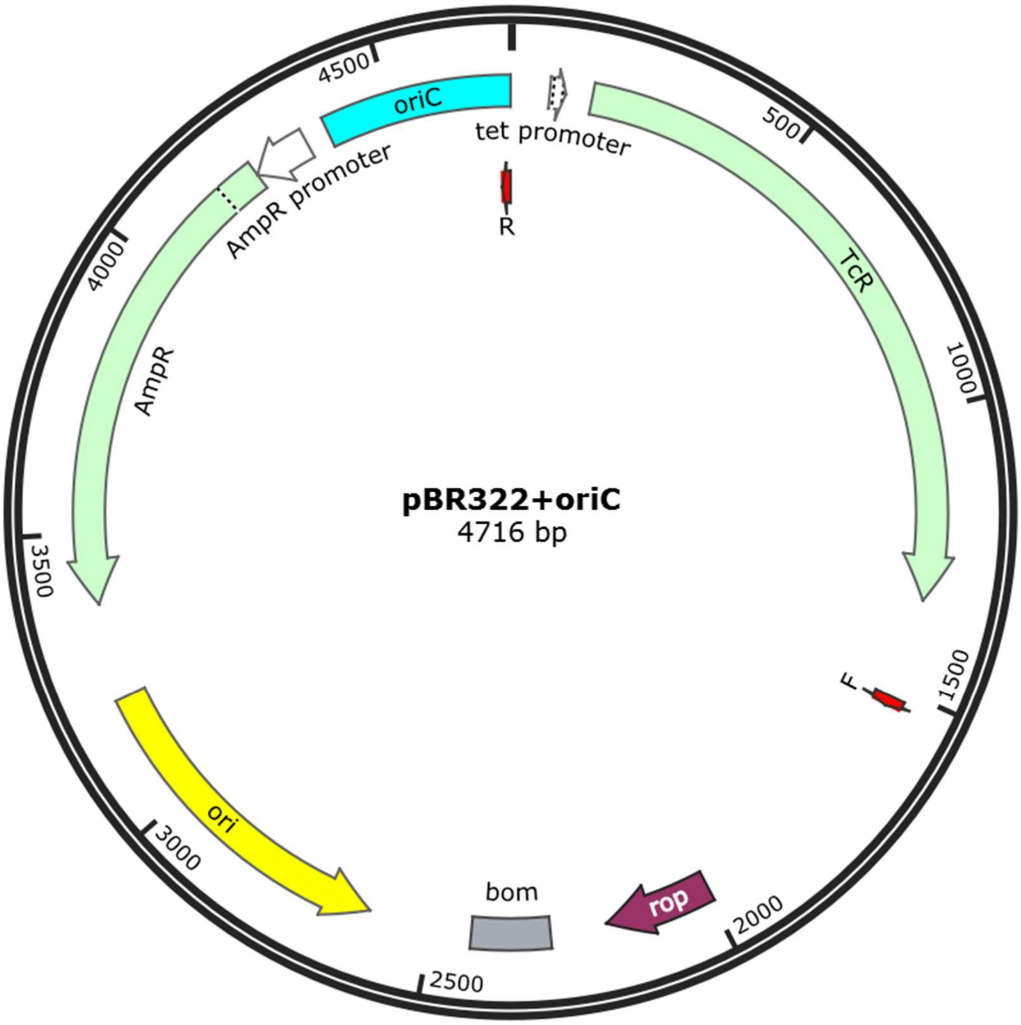

oriC cassette contained in OriCiro Cell-Free Cloning System (OriCiro Genomics, Tokyo, Japan) was subcloned into pBR322 downstream of the ampicillin resistance gene.

With the vector as a template, the region containing *rop*, *ori*, *AmpR*, and *oriC* cassette (*oriC*-*AmpR* cassette) was amplified by PCR. The sequence of primer used in this PCR amplification is as follows:

F 5'-CTGCTCTGATGCCGCATAG -3'

R 5'-GTGTCGGGGCTGGCTTAAC-3'

This PCR product was used as *oriC*-*AmpR* cassette in this paper.

Supplementary Methods 2. PCR amplification of *oriC-AmpR* cassette

oriC-AmpR cassette was amplified by PCR using primer pairs containing 60 nt overlapping sequences with the target sequence located at the 5'-side. PCR reaction was conducted as follows; initial denaturation at 94°C for 1 min, followed by 35 cycles of 94 °C for 30 s, 57 °C for 30 s, and 68 °C for 2 min. A final elongation step was 68 °C for 8 min. Afterward, electrophoresis on a 1% agarose gel at 100 V for 30 minutes was performed, and gel extraction was conducted using the QIAquick Gel Extraction Kit (QIAGEN).

The primers used in each experiment is as follows:

[chr5:149,503,216–149,513,393]

Forward Primer:

5'-AGGTTGGGCAGGCCCCCCAAATCAGGAGGGGCCGGGGAGAAATCAGCCTGCAAGCC AGATCTGCTCTGATGCCGCATAG-3'

Reverse Primer:

5'-TACATAGACTTATTCACGTTTGCAATGATCGCTCATTTCAACATTTTCGTTTGTGTAGAT GTGTCGGGGCTGGCTTAAC-3'

[chr10:79,309,011–79,336,568]

Forward Primer:

5'-GTTTTTCGGATGATTTTTTTCCTGGCTTGTCCACAACTTCTGGAAGCATCTTACTCAGA TCTGCTCTGATGCCGCATAG-3'

Reverse Primer:

5'-AGCAACAGATCAAAATTATTATTAAGACTGAAGTTACAATAAACAAAGCAAACAAAAG ATGTGTCGGGGCTGGCTTAAC-3'

[chr14:88,450,095–88,455,957]

Forward Primer:

5'-AAACCATAAACTTGGGCCACCTTTTGTCCAAACTTCCCATGTACCCTCAGTCACCAAG ATCTGCTCTGATGCCGCATAG-3'

Reverse Primer:

5'- TCACCTGTCACAAACTGTTAGTTAACACCTTTTAAGGATTTGAAAAGTATGAAAAATG ATGTGTCGGGGCTGGCTTAAC-3'

[chr2: 219,674,341–219,680,561]

Forward Primer:

5'-ACGGTCCTGGAGCTGGCTTTCTGTGAGCAATATTACCATGTGATCTAAATTTGCCCAGG TCTGCTCTGATGCCGCATAG-3'

Reverse Primer:

5'-CCCAACAAAGTAAAGTCTAATAAGACTGTCTAAGCTATGAATTCTGATTAATGACCAG TAGTGTCGGGGCTGGCTTAAC-3'

[chr6: 152,527,031–152,546,879]

Forward Primer:

5'-TGACCCCCATCCTGTCTCTCAATACCCATGAAGCCAGCAGTAACTGGACTTGGACCTC TGGCATGATCGTGCTCCTGTCG-3'

Reverse Primer:

5'-CGCATCACTTGCTCCAAGAGCGGGGCACTGGCCAGGTTCACGTGCATCTGAGGCCCT AAGGTGTCGGGGCTGGCTTAAC-3'

157

[chr6: 152,545,700–152,558,998]

Forward Primer:

5'-TTTATTTTATTTAAAACTGGAAAACACTTTTTAAAAGATCAGTTGAAGTCTTTTCCAGT ACTGCTCTGATGCCGCATAG-3'

Reverse Primer:

5'-AATGGAGTAGTCACAAAGTGGCCTTTGACAAGATAAACAGTTACCTCATGGAGGCCA GATGTGTCGGGGCTGGCTTAAC-3'

[chr13: 52,506,042–52,586,194]

Forward Primer:

5'-CGGCAAGAGTGAACTCCGCACCTGGAAAATCGATCCGCTGTGCGCAAAGGCCAGCCA ATGCTGCTCTGATGCCGCATAG-3'

Reverse Primer:

5'-ATAGGTTCCAGAGACATGCTGGTGGCATAGTCCGTTATCATATTTCAAGGTTCTCCCCC AGTGTCGGGGCTGGCTTAAC-3'

[chr6:152,599,149–152,671,977]

Forward Primer:

5'-AAAAAAAAATGAGCCTGCTCATCTCCGTGGACAAGATGAATACATACATATTCCCATG CTGCTCTGATGCCGCATAG-3'

Reverse Primer:

5'-GCTGGCCCACAGCTGAAAGTTGGCACACTGTTCACGTGCTCCTTCTAAGGTTTGCCA GGTGTGTCGGGGCTGGCTTAAC-3'

[chr6:152670346–152754123]

Forward Primer:

5'-GATGATCTGCACAGCGGGTAATCTTATCTGAGCAACATTGCACCCTCCCACCCTCCACA TCTGCTCTGATGCCGCATAG-3'

Reverse Primer:

5'-TTAAATCTATCAAGATAAGAACTTAATAATATGTATTTTTAGTAATCGTGTTTACCACGT GTGTCGGGGCTGGCTTAAC-3'

[chr3:184,074,010-184,081,131]

Forward Primer:

5’- GGACGGAAGTTAAGTAGCCCCGAGCGGGAGGCTGTGGCGGAAGTGGTCGCGTTAC
CGCTTCTGCTCTGATGCCGCATAG-3’

Reverse Primer:

5’- GACTTCCCTTGGGACTGACATCCTTCAGAGAGAGGGCAGCCAGCTGAACCTGCTCC
CTACGTGTCGGGGCTGGCTTAAC-3’

Supplementary Methods 3. The sequence of the primers used to confirm the variant

[Patient 1]

Variant 1

5’-ATGATGTCTCCATTTGGTAA-3’

Variant 2

5’-GGGCAAAAGTTAGGGCCATT-3’

[Patient 2]

Variant 1

5’-AGACCATCAGGCTCAGAGGA-3’

Variant 2

5’-GAGCCTGAAAGCTTCCAGCC-3’

[Patient 3]

Variant 1

5’-TTCTAGACGTGGTCCCATCTG-3’

Variant 2

5’-TTCAGGATCTGGAAGATTTG-3’

[Patient 4]

Variant 1

5’-TCCCATGGTCTTGGTGTTTT-3’

Variant 2

5’-CAAGCACATCTCCCAGACAG-3’

[Patient 5]

Variant 1

5’-AAGCTCAGCAGCATTGTAGGA-3’

Variant 2

5’-AGAACTGTCAGTCCACAATG-3’

SNP

5’-TCCTATGCTGCACAGGTCCT-3’

Patient 6

Variant 1

5’-ATTAGGGTAGGCCCCTGGT-3’

Variant 2

5’-AAGGGAAGAGGCACCTGAGT-3’

Supplementary Figure 1. RCR amplification and determination of the phase in Patients 2–7

a. RCR amplification of *SYNE1* locus (Patient 2)

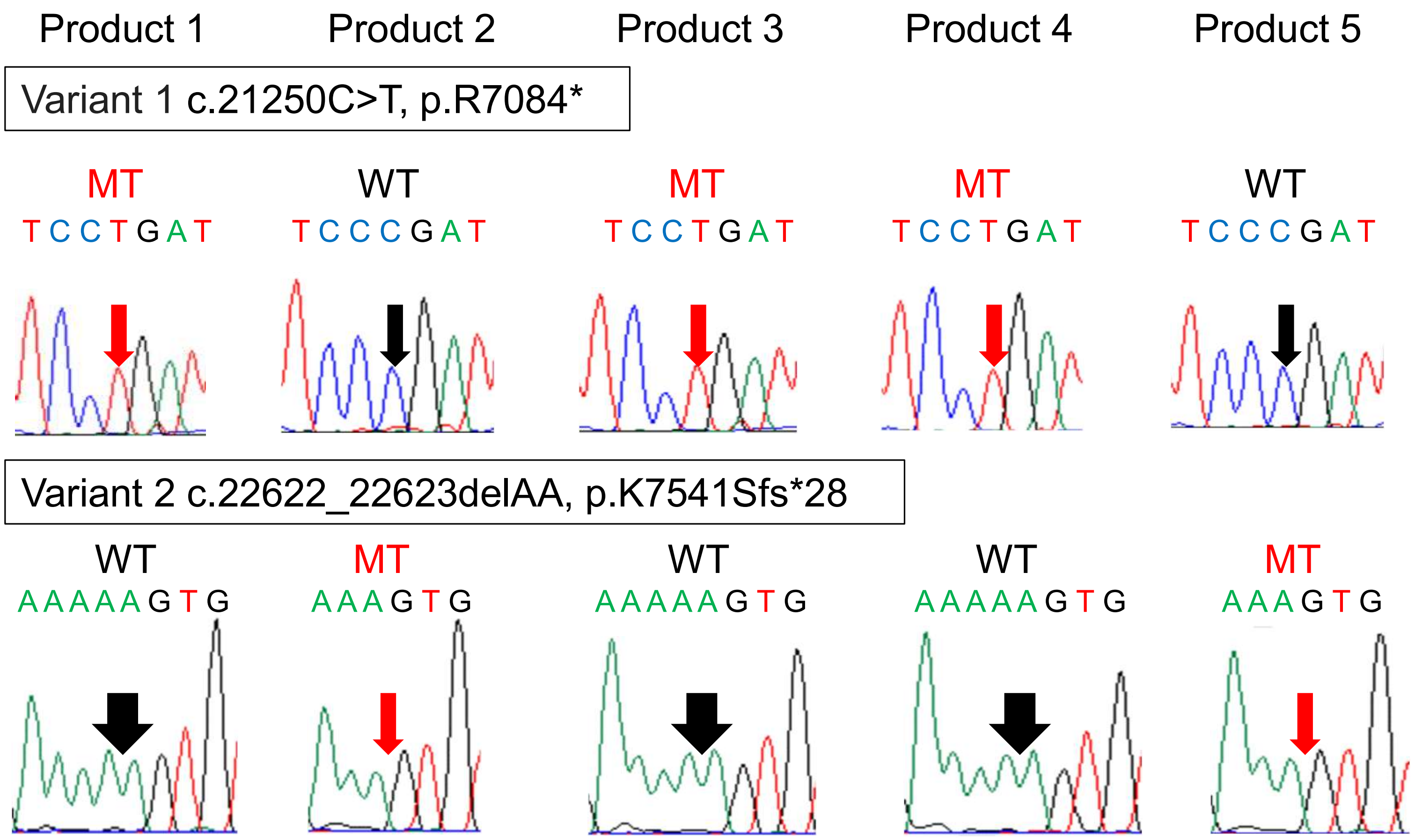

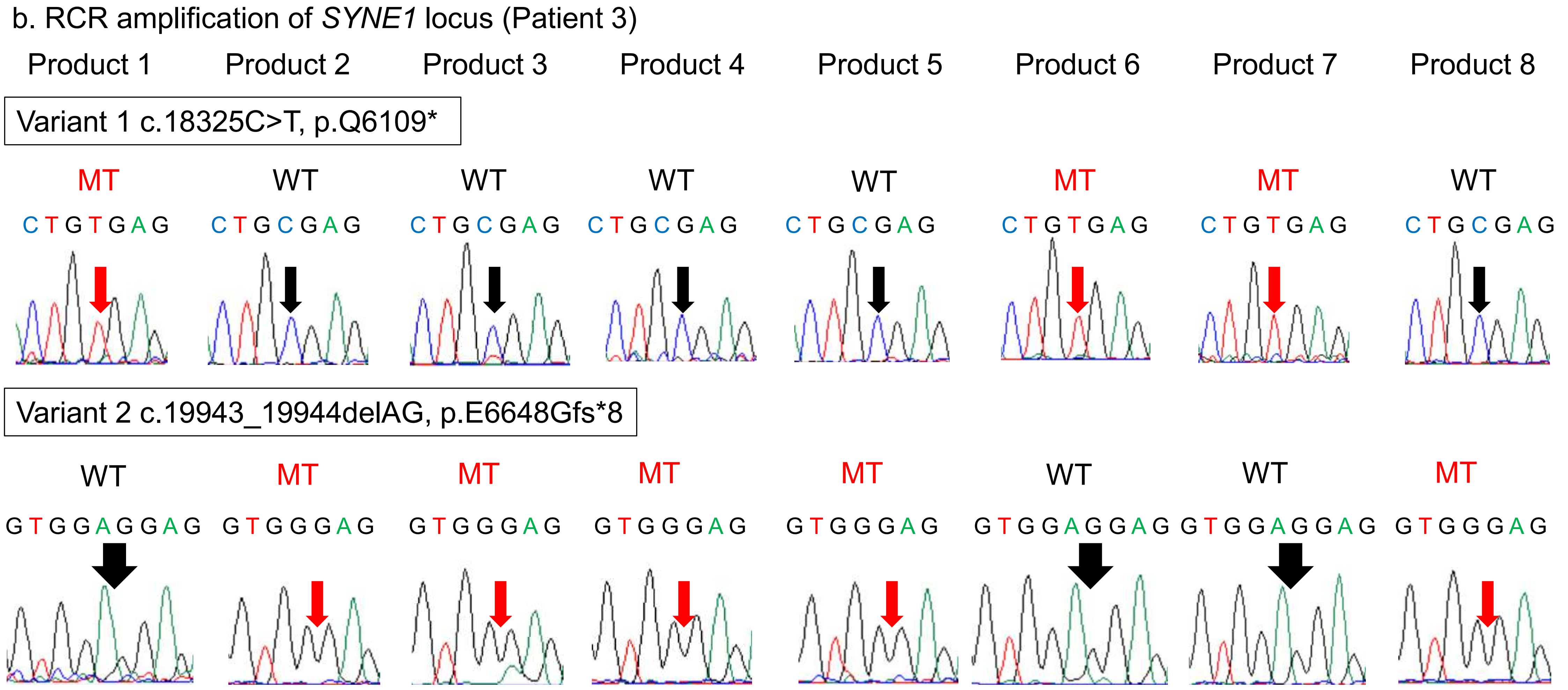
b. RCR amplification of SYNE1 locus (Patient 3)
Product 1
Product 2
Product 3
Product 4
Product 5
Product 6
Product 7
Product 8
Variant 1 c.18325C>T, p.Q6109*
MT
WT
WT
WT
WT
MT
MT
WT
C T G T G A G
C T G C G A G
C T G C G A G
C T G C G A G
C T G C G A G
C T G T G A G
C T G T G A G
C T G C G A G
Variant 2 c.19943_19944delAG, p.E6648Gfs*8
WT
MT
MT
MT
MT
WT
WT
MT
G T G G A G G A G
G T G G G A G
G T G G G A G
G T G G G A G
G T G G G A G
G T G G A G G A G
G T G G A G G A G
G T G G G A G

c. RCR amplification of *ATP7B* locus (Patient 4)

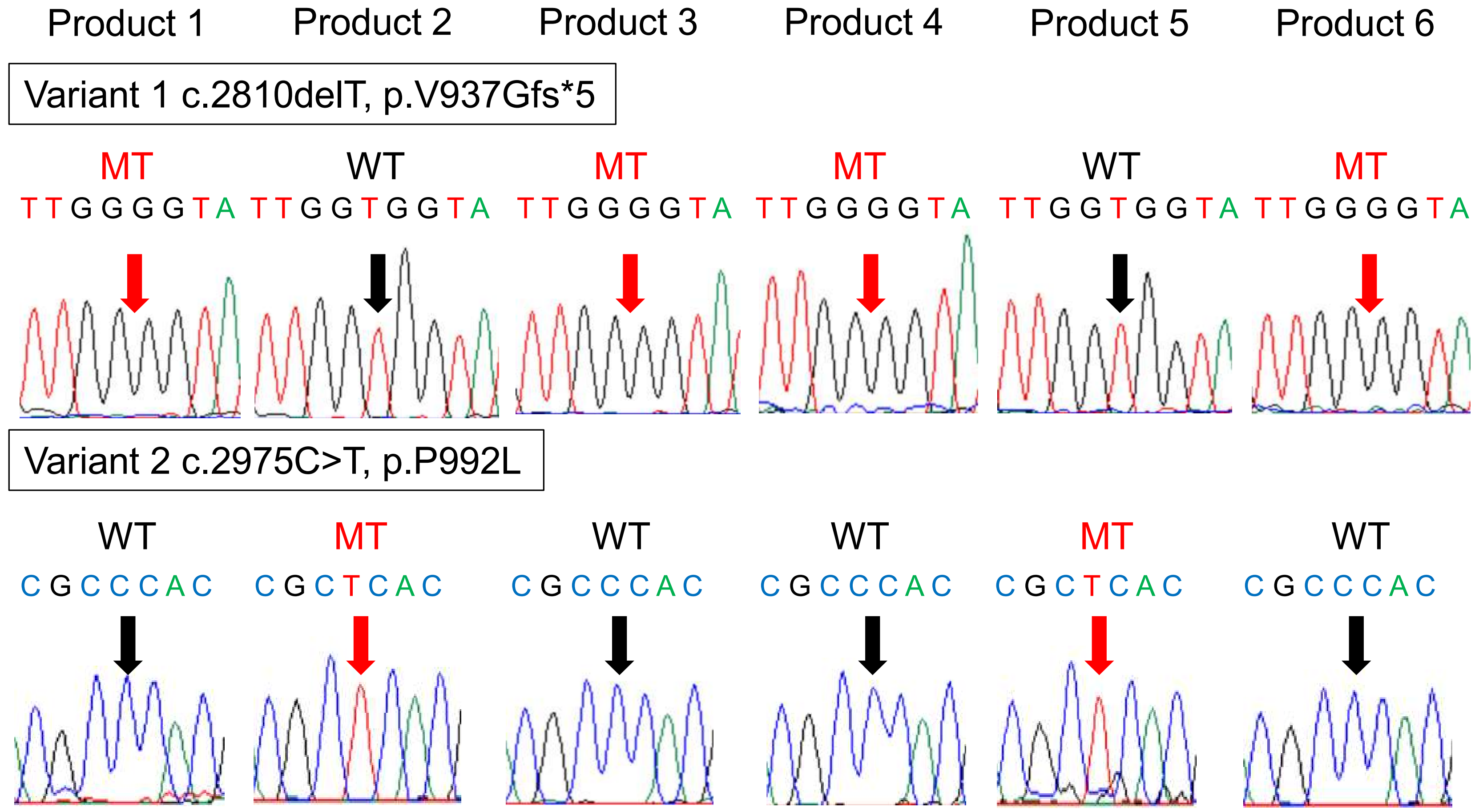

d. RCR amplification of *SYNE1* locus (Patient 5)

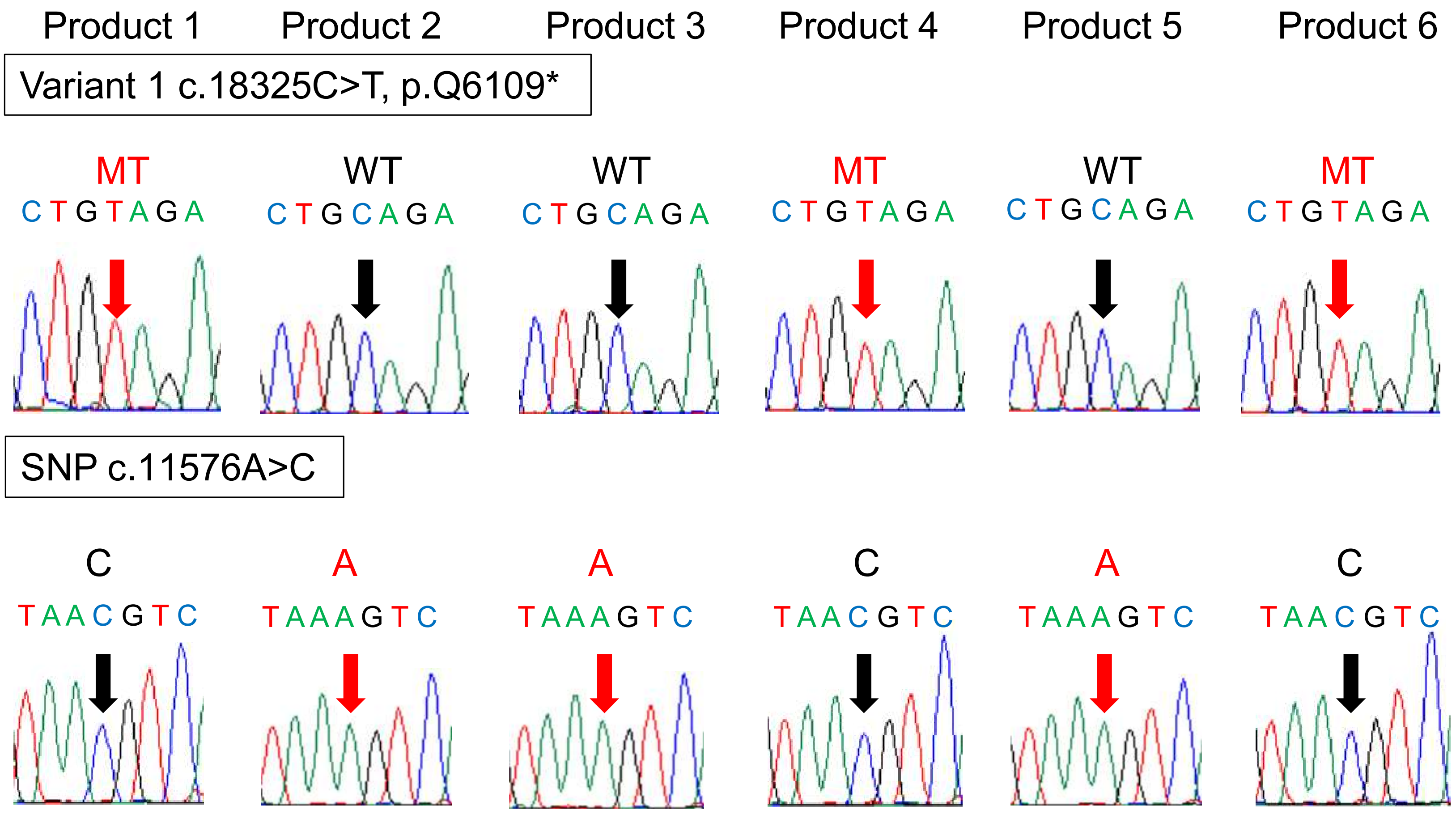

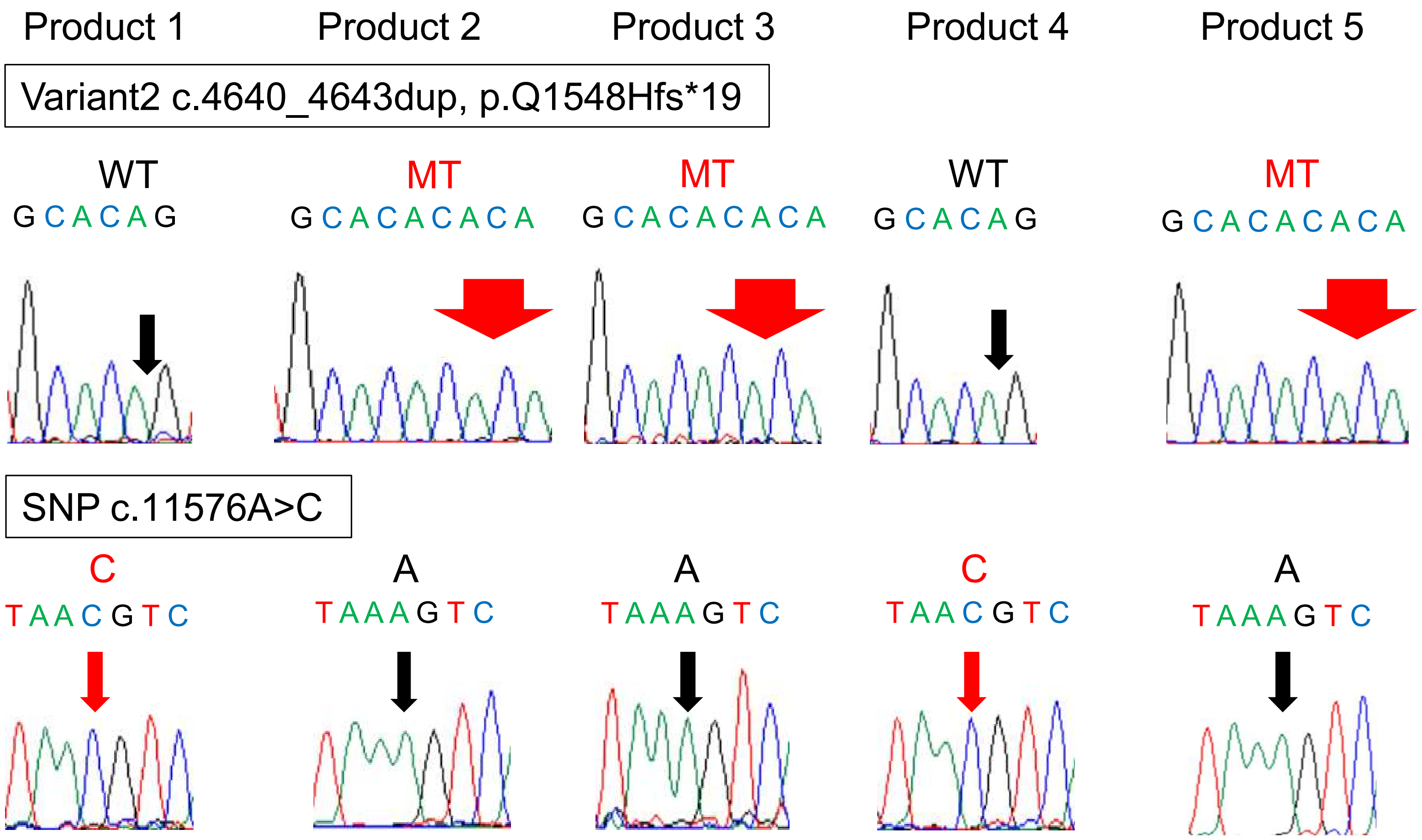
Product 1
Product 2
Product 3
Product 4
Product 5
Variant2 c.4640_4643dup, p.Q1548Hfs*19
WT
GCACAG
MT
GCACACACA
MT
GCACACACA
WT
GCACAG
MT
GCACACACA
SNP c.11576A>C
C
TAACGTC
A
TAAAGTC
A
TAAAGTC
C
TAACGTC
A
TAAAGTC

e. RCR amplification of *COQ4* locus (Patient 6)
gDNA:*oriC-AmpR*=1:100

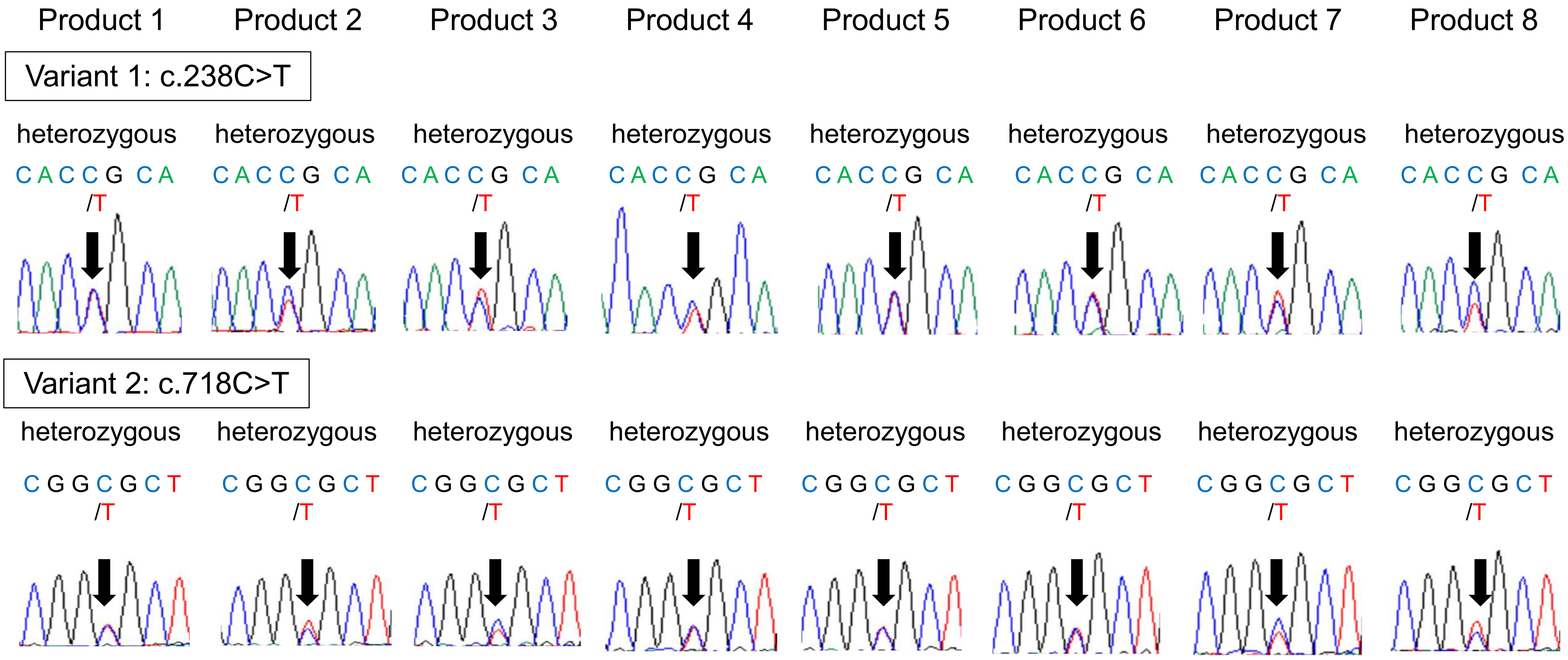

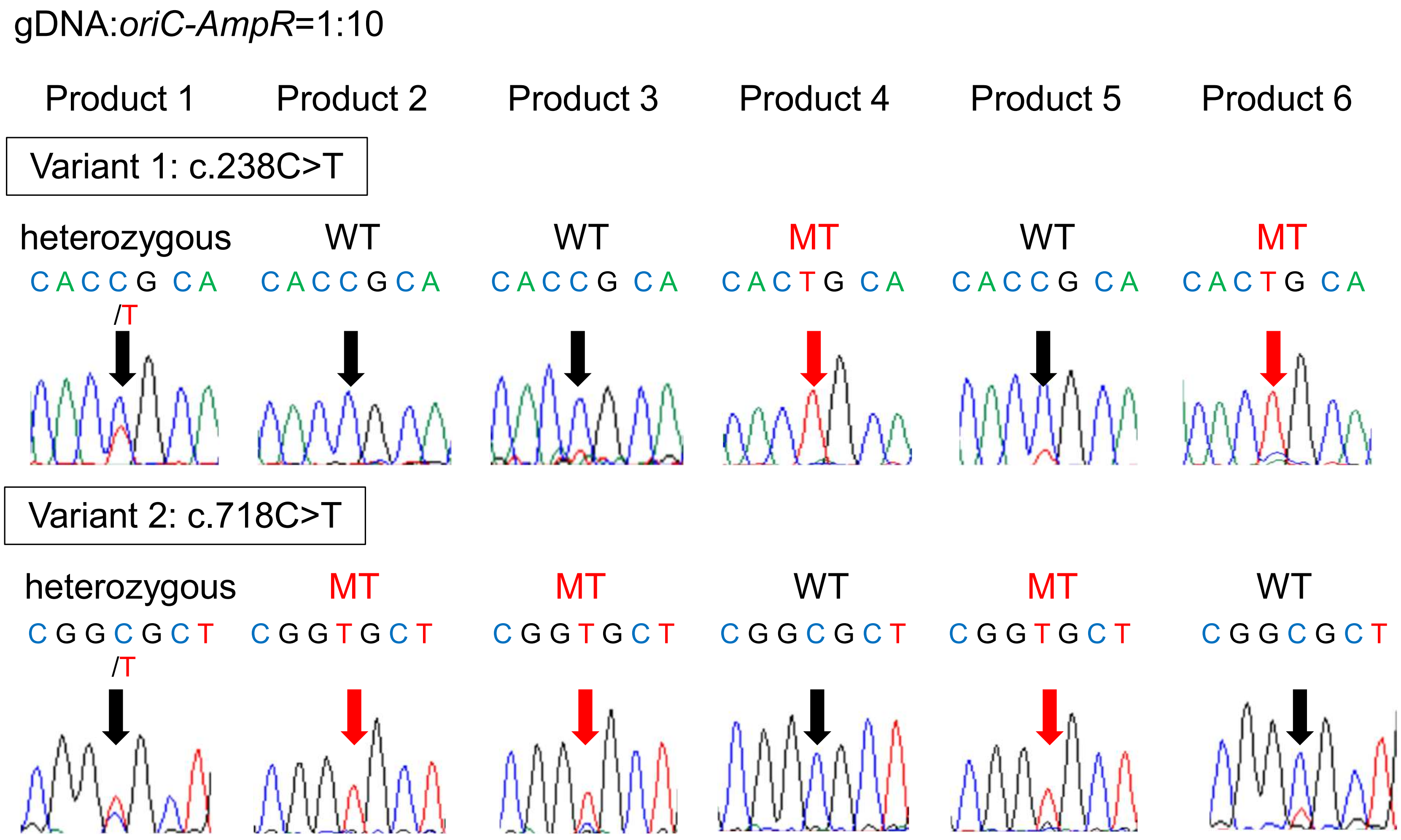
gDNA:oriC-AmpR=1:10
Product 1
Product 2
Product 3
Product 4
Product 5
Product 6
Variant 1: c.238C>T
heterozygous
WT
WT
MT
WT
MT
C A C C G C A
/T
C A C C G C A
C A C C G C A
C A C T G C A
C A C C G C A
C A C T G C A
Variant 2: c.718C>T
heterozygous
MT
MT
WT
MT
WT
C G G C G C T
/T
C G G T G C T
C G G T G C T
C G G C G C T
C G G T G C T
C G G C G C T

f. RCR amplification of *CLCN2* locus (Patient 7)

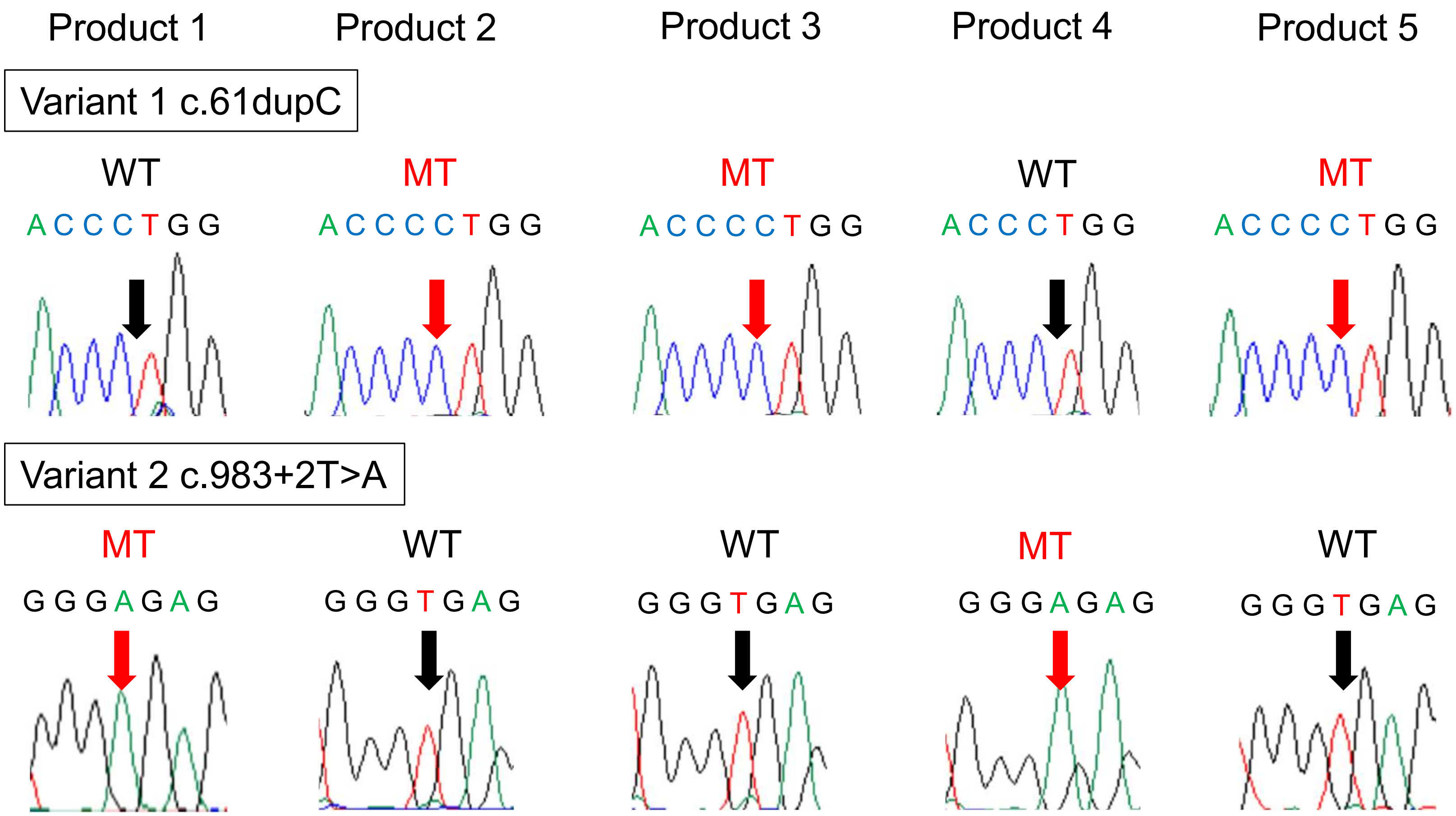

Supplementary Fig. 1. RCR amplification and determination of the phase in Patients 2 –7

a. Patient 2. In these five RCR products, only one allele is amplified.

b. Patient 3. In these eight RCR products, only one allele is amplified.

c. Patient 4. In these six RCR products, only one allele is amplified.

d. Patient 5. The region containing variant 1 (c.18325C>T, p.Q6109*) and heterozygous SNP (c.11576A>C) is amplified. In these six RCR products, only one allele is amplified. Similarly, the region containing heterozygous SNP (c.11576A>C) and variant 2 (c.4640_4643dup, p.Q1548Hfs*19) is amplified. In these five RCR products, only one allele is amplified.

e. Patient 6. In these eight RCR products, two alleles are amplified. Then, the molar ratio of gDNA to *oriC–AmpR* cassette is changed from 1:100 to 1:10. In these six RCR products, only one allele is amplified in five products.

f. Patient 7. In these five RCR products, only one allele is amplified.
